# Artificial intelligence, machine learning, computer-aided diagnosis, and radiomics: advances in imaging towards to precision medicine

**DOI:** 10.1590/0100-3984.2019.0049

**Published:** 2019

**Authors:** Marcel Koenigkam Santos, José Raniery Ferreira Júnior, Danilo Tadao Wada, Ariane Priscilla Magalhães Tenório, Marcello Henrique Nogueira Barbosa, Paulo Mazzoncini de Azevedo Marques

**Affiliations:** 1 Centro de Ciências das Imagens e Física Médica (CCIFM) da Faculdade de Medicina de Ribeirão Preto da Universidade de São Paulo (FMRP-USP), Ribeirão Preto, SP, Brazil.; 2 Escola de Engenharia de São Carlos da Universidade de São Paulo (EESC-USP), São Carlos, SP, Brazil.; 3 Faculdade de Medicina de Ribeirão Preto da Universidade de São Paulo (FMRP-USP), Ribeirão Preto, SP, Brazil.

**Keywords:** Artificial intelligence, Machine learning, Computer aided diagnosis, Radiomics

## Abstract

The discipline of radiology and diagnostic imaging has evolved greatly in recent years. We have observed an exponential increase in the number of exams performed, subspecialization of medical fields, and increases in accuracy of the various imaging methods, making it a challenge for the radiologist to “know everything about all exams and regions”. In addition, imaging exams are no longer only qualitative and diagnostic, providing now quantitative information on disease severity, as well as identifying biomarkers of prognosis and treatment response. In view of this, computer-aided diagnosis systems have been developed with the objective of complementing diagnostic imaging and helping the therapeutic decision-making process. With the advent of artificial intelligence, “big data”, and machine learning, we are moving toward the rapid expansion of the use of these tools in daily life of physicians, making each patient unique, as well as leading radiology toward the concept of multidisciplinary approach and precision medicine. In this article, we will present the main aspects of the computational tools currently available for analysis of images and the principles of such analysis, together with the main terms and concepts involved, as well as examining the impact that the development of artificial intelligence has had on radiology and diagnostic imaging.

## INTRODUCTION

The discipline of radiology and diagnostic imaging has evolved greatly in recent years. Radiological imaging can be extremely complex, and it is recognized that the analysis of exams that produce hundreds of images, such as computed tomography (CT) and magnetic resonance imaging (MRI), poses challenges, even for experienced specialists^(1-3)^. Those challenges have increased in recent years, with the exponential increase in the number of exams performed, subspecialization of medical fields, and increased accuracy of imaging methods, making it difficult for radiologists to “know everything about all exams and regions”. In addition, imaging exams are no longer only qualitative and diagnostic, having begun to provide quantitative information on disease severity, as well as identifying biomarkers of prognosis and treatment response^(4-6)^. Those changes have been especially prominent in oncology, showing that the information provided by imaging studies can go far beyond determining whether a lesion is benign or malignant, now being able to indicate the histological type of the tumor, staging, presence of mutations, chance of treatment response, risk of recurrence, and expected survival^(7-9)^.

Computer-aided diagnosis (CAD) systems have been developed with the objectives of improving the accuracy of exams, increasing consistency in interpretation of images, helping the prognostic evaluation, and supporting the therapeutic decision-making process. Although such tools have enormous potential, there are still limitations to their use in routine clinical practice. With the advent of artificial intelligence and “big data”, we are moving toward reducing those limitations, homogenizing and expanding the use of CAD tools in daily routine of physicians, making each patient unique, and leading radiology toward the concepts of a multidisciplinary approach and precision medicine^(10-14)^.

In this article, the main aspects of the computational tools currently available for image analysis will be discussed, as will the impact of the development of artificial intelligence and the role of imaging in precision medicine. Table 1 presents the main terms that will be used throughout this text, together with a brief definition of each.

**Table 1:** Key terms that have been used throughout the text, together with their short definitions.

Term	Definition
Machine learning	Field of computer science that involves the evolution of pattern recognition systems, allowing computers to learn from errors and predict outcomes.
Deep learning	Branch of machine learning that attempts to model large amounts of data using multiple processing layers.
Features of the image	Image characteristics used in computational analysis, classified into three groups: gray levels, texture, and shape.
Big data	Set of data and information that can be stored and analyzed by modern computational analysis tools-large in volume, speed, and variety.
Computer aided diagnosis/detection	Medical diagnosis/detection using the results of automated quantitative image analyses as a "second opinion".
Content based imaging retrieval	System that enables images or exams to be retrieved from information based on the pictorial content of a reference image or exam.
Artificial intelligence	Human-like intelligence displayed by machines or computer programs.
Precision medicine	Model of medicine that proposes the personalization of health care, with individualized diagnoses and treatments for each patient.
Radiomics	Massive extraction of measurable imaging data and their integration into multidisciplinary predictive models for the management of the diagnosis, treatment, and prognosis of patients.
Artificial neural network	Machine learning method based on the human central nervous system, with computational models made up of layers, each layer being composed of neurons.
Convolutional neural network	Class of artificial neural network designed to require as little preprocessing as possible..

## PRINCIPLES OF COMPUTER-AIDED IMAGE ANALYSIS IN MEDICINE

**Figure 1:**
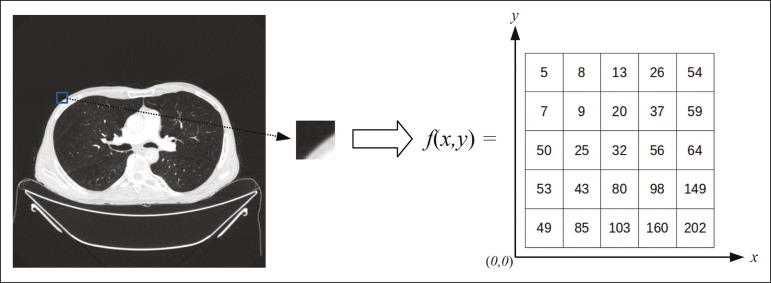
Representation of the function (matrix) of a gray-scale digital image (axial slice of a chest CT).

**Digital medical imaging** is an f (x, y) function in a spatial coordinate-partitioned gray scale that can be represented by a matrix in which the intersection of each row and column identifies a single point (pixel) within an image. As depicted in Figure 1, the value of each pixel in the matrix identifies the gray level at that point (x, y) on a scale of integer values that represent black (the lowest value), white (the highest value), and shades of gray (intermediate values). With CT and MRI, images can be acquired volumetrically, in form of a volume of parallel, evenly spaced slices, so that a point in the image represents a voxel, with a “height” equal to the thickness of the slice^(15-17)^. The processing and analysis tools work with this matrix of numerical values that represent the image^(18)^.

There was a major technological revolution in radiology in the late 1990s and early 2000s, when filmless radiological environments that were fully digitized and integrated with other information systems were established within a model known as a digital hospital^(19)^. The foundation of filmless radiology is the picture archiving and communication system (PACS), which is a mean of receiving images in the standard digital imaging and communication in medicine (DICOM) format from the various acquisition devices (conventional radiography, ultrasound, CT, and MRI), making them available for specialists or other computer systems to access, and storing them in an organized way in large databases^(20)^. The integration of the PACS with the clinical information systems known as hospital information systems (HIS) and radiology information systems (RIS) has also enabled the development of various CAD models^(21,22)^.

**Figure 2:**
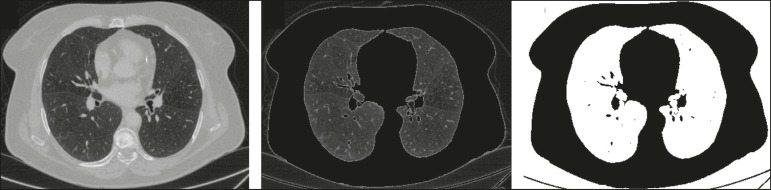
Semiautomated segmentation of the lung on a CT scan of the chest with 256 gray levels and a user-defined threshold of 115 Hounsfield units: the original CT image (image on the left) is thresholded (to detect the edges) and transformed into a binary image (to separate the lungs).

**Figure 3:**
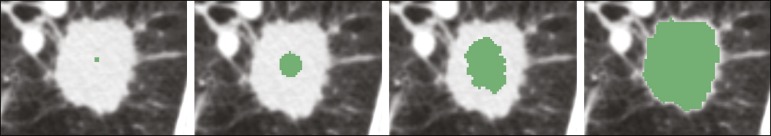
Semiautomated segmentation, with region growing, of a neoplastic pulmonary nodule on chest CT after placement of a user-defined seed pixel (point at the center of the nodule in the first image on the left).

One of the key steps in image processing and analysis is **segmentation**, which often represents major challenges. The main objective of segmentation is to divide the image into parts that represent normal anatomy and those that are abnormal; to segment is to separate tissues and structures on the basis of their anatomical characteristics^(23)^. In some cases, segmentation can be simplified by using shapes that are predefined (circles or rectangles) or outlined in the image, representing a region of interest (ROI) that does not necessarily encompass all of the tissue, structure, or organ. Techniques for the segmentation of images are generally based on basic properties of gray levels, discontinuity (edges), or similarity (after setting thresholds or using a region-growing algorithm), as shown in Figures 2 and 3. The segmentation process can be manual, semiautomated (the user intervenes at some point in the process), or fully automated^(24)^.

The process of **feature extraction** consists in calculating numerical values (descriptors) that represent the visual content of an image. Features are obtained by executing algorithms known as feature extractors. Feature extraction algorithms perform quantitative imaging procedures such as histogram construction, texture classification, shape recognition, and contour recognition, as well as area and volume estimates. After features have been extracted by these algorithms, the values are stored in a feature vector.

**Figure 4:**
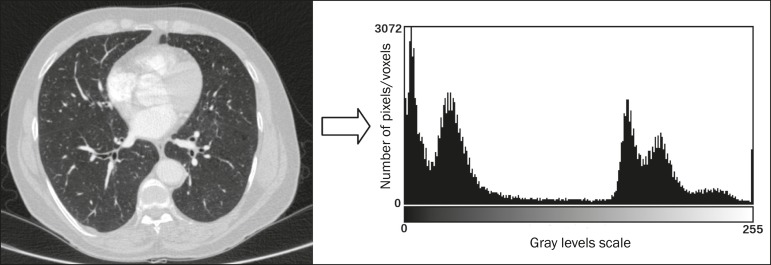
Example of the histogram of an axial CT scan of the chest with 256 gray levels. The histogram shows the distribution of pixels or voxels according to the gray levels (or Hounsfield units, if necessary).

**Figure 5:**
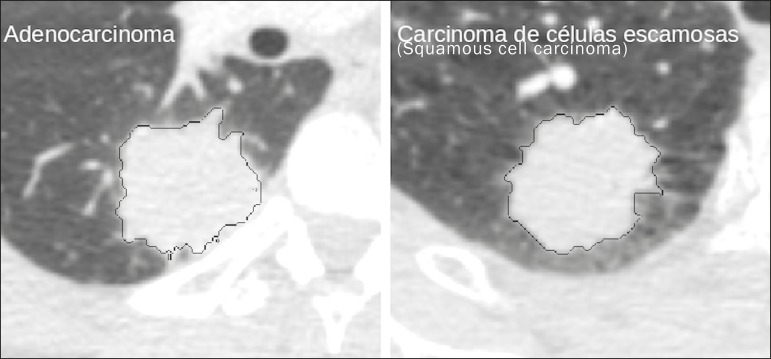
Segmentation of pulmonary nodules on chest CT in two different patients, both nodules having irregular contours and being in proximity with adjacent vessels or chest wall, factors that make it difficult to segment the edges of lesions properly. In such cases, texture analysis can facilitate proper segmentation.

Features are basically classified into three main groups: gray level, texture, and shape. The extraction of ***gray-level*** features is the most widely used technique, which can be performed directly or by analysis of the histogram. As can be seen in Figure 4, a histogram is a description of the number of gray levels present in the image, and that calculation involves only the pixel/voxel count with the gray-scale intensities^(3,7,25)^. However, using only gray-level descriptors or histograms does not provide information on the spatial distribution of the content of an image, which can be determined by analyzing ***texture*** features^(24,26)^. In some images, regions that have similar pixels/voxels are distinguishable because of their different textures (Figure 5). Texture features have become particularly important because they can reflect the details of a lesion identified in an image^(1,2)^. ***Shape*** features describe the edge of the image and the geometric features extracted from the segmented object, such as its contours, junctions, curves, and polygonal regions^(2,27)^. Characterizing object shapes quantitatively is a complicated task because it depends on efficiency of the segmentation algorithms. Lesions seen in radiological images of the lung, for example, often have adjacent opacities or structures such as vessels and the mediastinum (Figure 5), which can lead to poor segmentation and, consequently, poorer characterization by shape features.

**Selection of relevant features** is another important step, given the wide range of features that can be extracted from the image. In many cases, some characteristics are irrelevant for a given analysis or data are redundant, introducing noise or inconsistencies into the feature vector^(28)^. Therefore, it is necessary to select the most relevant characteristics according to clinical investigation class (diagnosis or outcome). Various algorithms have been created in order to reduce spatial dimensionality of the vector and can be classified into three main types: filter, wrapper, and embedded. Most algorithms use machine learning (ML) resources, and some perform feature selection by using artificial neural networks (ANNs), decision trees, and random forests^(11)^.

## IMAGE CLASSIFICATION, MACHINE LEARNING, AND DEEP LEARNING

Image classification typically involves defining the image within a pre-established category, such as normal versus pathological. One of the most widely studied areas in artificial intelligence and image classification is **machine learning**. Machine learning allows the identification of patterns seen in previous cases and experiments, as occurs with human intelligence^(29)^.

Machine learning methods have been applied to classifying images acquired with various imaging modalities, using a variety of features, for various diseases, and with tools such as CAD and radiomics^(25,29,30)^. Developing a machine learning method involves creating a training function for a dataset (the feature vectors, in the case of image classification) and making use of logical inference. When classes (diagnoses or clinical outcomes) for final decision of the model are pre-established, the training process is supervised. When there is no defined class, the process is unsupervised. In the latter case, the algorithm is aimed at the formation of clusters of similar samples (“exams with a similar pattern”), which may or may not be related to a known condition or disease^(31,32)^.

**Figure 6:**
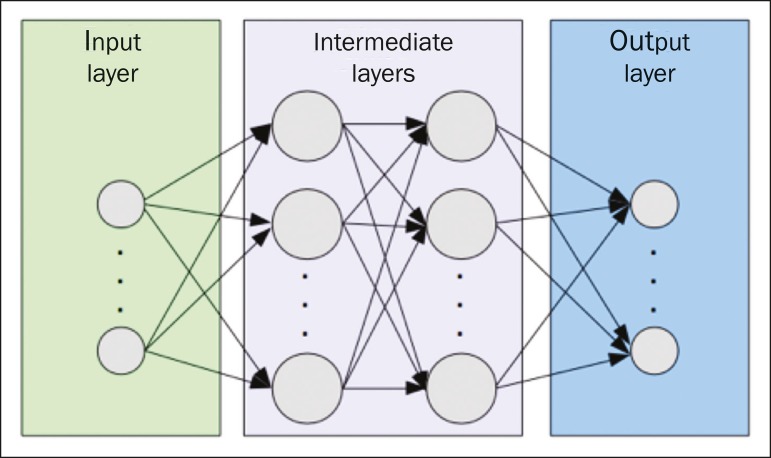
Architecture of a multilayer ANN. The input layer receives the feature information. The output layer represents classes or outcomes (e.g., normal versus pathological).

One of the most traditional machine learning methods involves ANNs, which are widely used in image classification tasks^(25,29,30)^. These neural networks were projected with the structures of the human central nervous system as a reference^(33)^ , the mathematical models being described in the form of layers, each layer consisting of N neurons. The best known type of ANN is the multilayer perceptron (MLP). Traditionally, the MLP network has an input layer (whose neurons correspond to features of the image), an output layer (whose neurons correspond to classes/outcomes), and a set of intermediate hidden layers (whose neurons correspond to fit points of the activation functions), as depicted in Figure 6.

**Figure 7:**
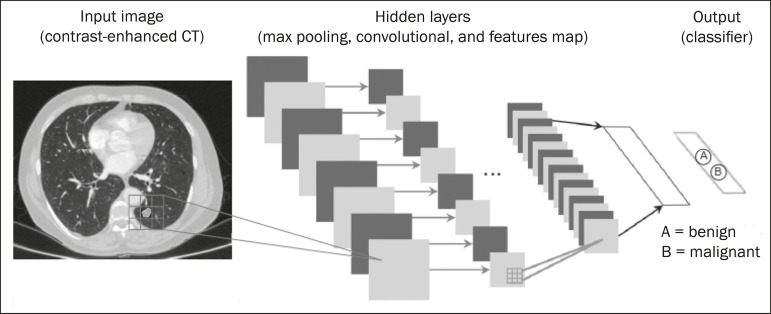
Chest CT image with a pulmonary nodule as input into a CNN for analysis using deep learning technique.

One of the areas of artificial intelligence that has been gaining attention in the scientific community most recently is **deep learning** ^(16)^. Traditional machine learning methods have limitations in data processing, mainly related to the need for segmentation and development of feature extractors to represent images and serve as input for the classifiers^(34)^. Therefore, researchers began to develop algorithms that integrated the processes of feature extraction and image classification within the ANN itself. Therefore, in deep learning technique, the need for preprocessing or segmentation is minimized. However, the method also has disadvantages, such as the need for a very large set of images (hundreds to thousands); greater dependence on exam quality and clinical data; and difficulty in identifying the logic used (“processing black box”). The most widely known method of deep learning in medicine is that involving a convolutional neural network (CNN). A CNN is basically composed of three types of layers^(35)^: the first (convolutional layer) detects and extracts features; the second (pooling layer) selects and reduces the amount of features; and the third (fully connected layer) serves to integrate all of the features extracted by the previous layers, typically by using an MLP-like neural network to perform the final image classification, which is given by the prediction of the most likely class (Figure 7).

**Figure 8:**
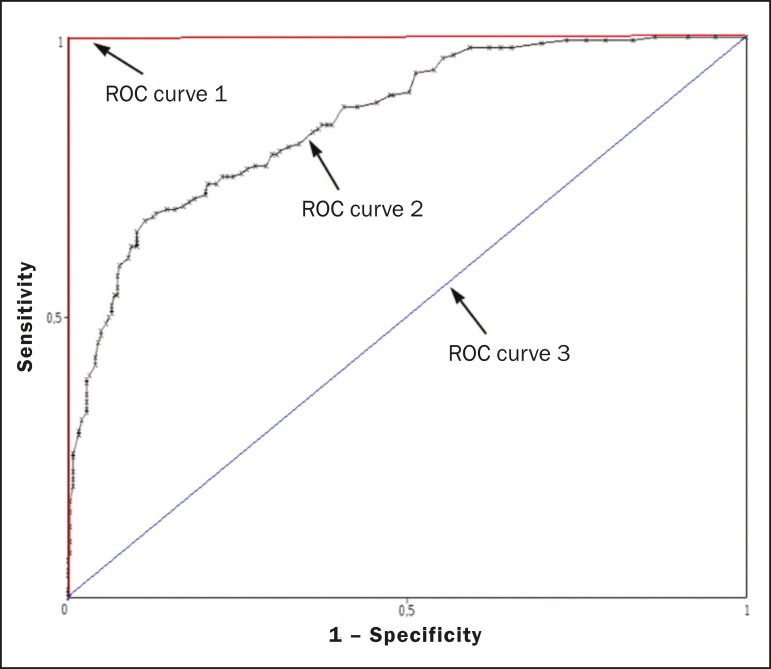
Example of ROC curves: curve 1 represents a test with perfect performance (AUC = 1.0); curve 2 represents a test with intermediate performance (AUC = 0.75); and curve 3 represents a random test (AUC = 0.50).

Another important step in the machine learning process is ***validation and performance assessment***. Given a set of images, a machine learning classifier must use at least two different subsets to perform algorithm ***training*** and predictive model ***validation***. A widely used strategy in radiology is cross validation. In cross validation, the samples are separated into N subsets^(13,36)^ : N − 1 for training; and 1 for testing. Another strategy, which reduces the risk of model overfitting, is based on three subsets^(29,37)^: one for training, one for validation, and one (independent subset) for testing only. Performance is typically evaluated by calculating the accuracy, sensitivity, specificity, and area under the receiver operating characteristic (ROC) curve for the method in question. An area under the curve (AUC) closer to 1 (on a scale from 0 to 1) indicates greater the accuracy of the method (Figure 8).

Tools that employ artificial intelligence, machine learning, and deep learning can be used in different ways to analyze images. In the field of radiology and diagnostic imaging, such tools have been applied primarily in CAD, content-based image retrieval (CBIR), and radiomics/radiogenomics.

## COMPUTER AIDED DETECTION

**Figure 9:**
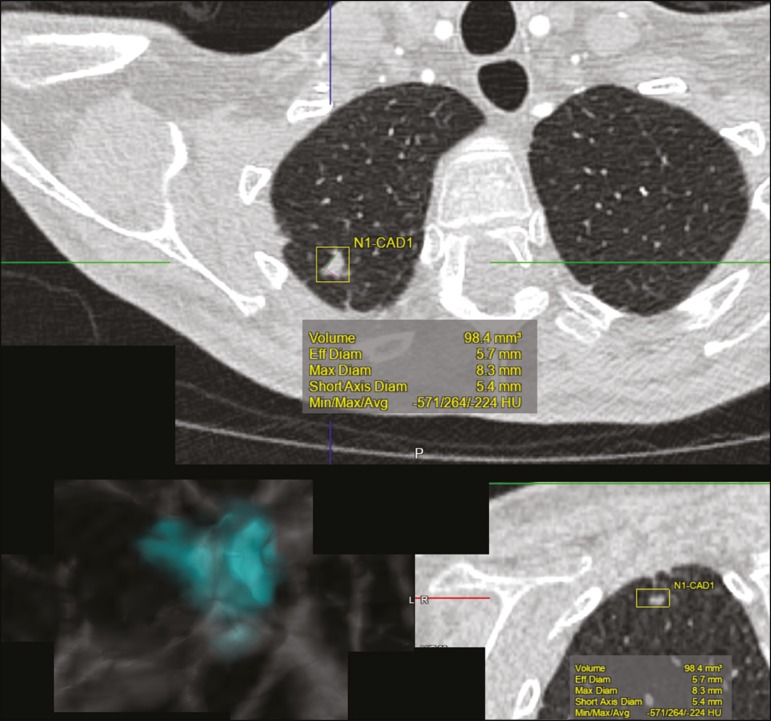
Example of a CAD tool for detection and analysis of pulmonary nodules. The program not only indicates the presence of a right apical pulmonary nodule but also provides quantitative and three-dimensional information regarding that nodule.

Tools of CAD were initially developed to aid in the interpretation of radiological findings and early identification of diseases, especially breast and lung cancer^(7,10)^.The aim of CAD is to improve the accuracy and consistency of diagnostic imaging by using suggestion of a response (“second opinion”) provided primarily by image processing, computer vision, and machine learning techniques^(1,3,38)^. Historically, the first CAD systems emerged in the late 1980s and were based on the processing of digitized radiography films. They were fundamentally designed to work as a second reading of exams in population-based cancer screening programs (helping detect nodules and microcalcifications on mammograms). Subsequently, similar systems were used in order to detect and classify pulmonary nodules on conventional radiographies and CT scans of the chest. More recently, these systems have been used to facilitate the diagnosis of Alzheimer’s disease in nuclear medicine exams. In these traditional CAD models^(25,29)^, the idea is for the second reading to be done by the computer rather than by a second radiologist (Figure 9).

With the development of artificial intelligence and new machine learning tools, auxiliary diagnostic systems have expanded greatly and have been used in many different tasks, with all medical imaging modalities. We can cite, for example, the surprising number of presentations (seminars, abstracts, and oral presentations) related to such systems at the 2018 Annual Meeting of the Radiological Society of North America, currently the largest radiology conference in the world, in terms of number of participants and number of presentations. That year, there were 237 registered events related to artificial intelligence, machine learning, or deep learning (https://rsna2018.rsna.org/program/index.cfm). Examples include the following: automated detection of pulmonary nodules, pneumonia, pneumothorax, and pleural effusion on conventional chest radiography; detection and quantification of emphysema, estimation of lung nodule malignancy risk, chance of local invasion by lung cancer, and cardiovascular risk on chest CT; and automated analysis of cardiac function on cardiac MRI. Therefore, possibilities for developing computerized auxiliary diagnostic tools have become almost unlimited^(39,40)^.

## CONTENT-BASED IMAGE RETRIEVAL

The concept of CBIR refers to the search for images whose contents are similar to a reference case by using information derived from the images themselves, represented by their intrinsic content (feature vector), rather than associated texts (reports) or external annotations^(41)^. Because it has potential for clinical application, as well as for application in teaching and research, CBIR has been described as one of the most promising computational tool^(42,43)^. It can be a very useful tool in daily clinical practice, because it can aid radiologists in diagnostic interpretation of exams, or increase their confidence level, because it uses a decision model based on similar exams^(22)^. Currently, the most common scenario is that the physician, in a case of a diagnostic uncertainty, searches the Internet via a browser or on specialized radiology sites for similar texts or cases, using keywords or using their diagnostic suspicion (e.g., Google searches for “lung nodule on chest CT” or “pulmonary hamartoma”). The use of CBIR takes the place of such strategies and is more effective, rapidly providing physicians with cases similar to that represented by the image for which they seek definition. Therefore, whereas CAD systems perform image classification tasks, generally providing a single response (lesion or no lesion, benign or malignant), CBIR systems perform image searches for similarity, providing a set of cases similar to an unknown case indicated by the physician.

## RADIOMICS AND RADIOGENOMICS

The suffix -omics was first used in the field of molecular biology, to describe the detailed characterization of molecules such as DNA (genomics) and proteins (proteomics). Radiomics has been described as an extension of CAD that associates the quantitative characteristics (features) of images with patient data and clinical outcomes, not only allowing the diagnosis to be made but also providing information regarding the prognosis and treatment response^(7,25)^. In view of recent advances in targeted treatment and immunotherapy, particularly in the treatment of malignancies, the need for a robust approach to imaging analysis has become clear, and radiomics has the potential to provide this in a noninvasive, rapid, timely, and affordable manner^(12)^. Radiomic analysis is a process of massive extraction of features from tens to hundreds of exams, inserting these features into databases with patient clinical information, allowing them to be shared and analyzed^(44)^.

The volume of health data has been growing at a rapid pace in recent years, characterizing what some authors call the “big data era” of health, and those electronic data are available in large quantities in information systems of large hospitals and other health care centers^(45)^.

Given the large number of features and numerous processing possibilities, some authors have started to develop and suggest the use of a **radiomic signature**, in which the most significant features of different categories are chosen, analyzed, and tested with accurate protocols and reproducible algorithms^(46)^.

In the medical literature, there are already many studies using radiomics in different types of imaging studies to evaluate different diseases. Most of those studies are related to oncology, such as the study of lung and kidney neoplasms on CT and positron-emission tomography/CT scans, as well as prostate cancer, breast cancer, glioma, and hepatocellular carcinoma on MRI^(7,14,36)^.

**Figure 10:**
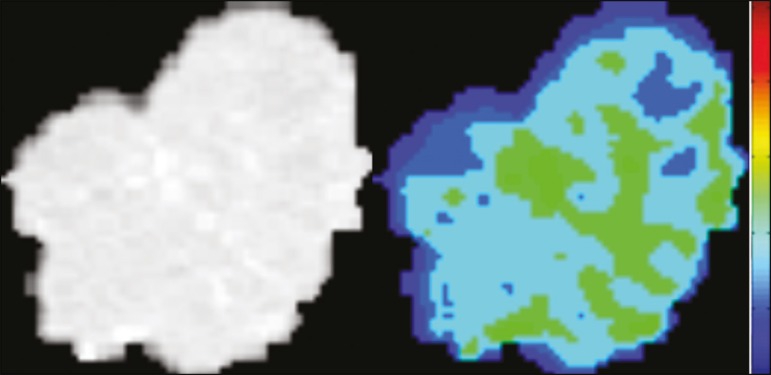
Quantification of the heterogeneity of a pulmonary adenocarcinoma on a CT scan of the chest by radiomic/radiogenomic evaluation. Color scale refers to a feature extracted from the image, reflecting tissue and genetic subregions of the tumor.

When the focus of radiomics is the study of correlation between radiological and genomic patterns (a set of genes), the process is known as radiogenomics^(47)^. Many studies have shown that imaging features are significantly associated with patterns of gene expression and genetic mutations, demonstrating that radiogenomic analysis can identify different biological mechanisms by means of mathematical and computational devices, enabling the decoding of disease phenotypes by noninvasive methods^(48,49)^. Radiogenomics has also been used in studies that analyze tumor heterogeneity (i.e., the presence of multiple tissue and genetic subregions within the same tumor), which is related to disease recurrence and treatment resistance. Radiogenomics is able to quantify the spatial complexity of the tumor and identify these phenotypic/genotypic subregions^(50)^ , as shown in Figure 10.

## CONCLUSION

Radiology has undergone significant advances due to the technological revolution that is taking place in the world. First, there was the digitization of radiological environments. Then, evolution of computer vision techniques and artificial intelligence led to the development of auxiliary diagnostic systems. More recently, maturation of computational models has provided support to the clinical decision-making and prognostic prediction processes. In this paper, we have presented and discussed the main concepts related to computer-aided image analysis, including aspects of artificial intelligence applied to precision medicine.

We believe that artificial intelligence, machine learning, computer-aided diagnosis, and radiomics will change the way radiologists and other imaging specialists work and will likely, in the very near future, change the perspective that everyone in the health care field has on their work. However, some people fear that radiologists and other specialists will be completely replaced by computer algorithms. Although simple tasks and exams (e.g., the evaluation of scoliosis or bone age on conventional radiographies) might be performed and interpreted entirely by such algorithms, the role of the physician in verifying/validating the outcome, making the clinical-epidemiological correlation, and determining the best treatment regimen are unlikely to be threatened. Of course, there are also ethical and legal issues related to medical exam liability.

Artificial intelligence will certainly help “reduce the backlog” of exams; shorten the time to action in urgent cases; streamline interpretation and reporting; increase diagnostic confidence; make image analysis more objective and reproducible; offer more reliable prognostic information; assist in the teaching and learning of imaging techniques; and lead radiology definitively toward the concepts of precision medicine and multidisciplinary patient assessment. In practice, it is thought that the first change will be that the radiologists of today, who mostly use a workstation with two computer screens (one with the imaging tool, the other with the system for emitting the report and access to clinical and radiological data) will begin to work with three screens, the third being one that includes the artificial intelligence analysis. Rather than fearing what the future will bring, radiologists need to prepare, learn, and adapt, because change is inevitable.
