# ERRATUM

**DOI:** 10.1590/0100-3984.2022.er-1-en

**Published:** 2022

In the article **Artificial intelligence, machine learning, computer-aided diagnosis, and radiomics: advances in imaging towards to precision medicine**, DOI: http://dx.doi.org/10.1590/0100-3984.2019.0049, published in **Radiologia Brasileira**, 52(6):387-396, on page 387, line 7:

*where it reads:*

**Marcello Henrique Nogueira Barbosa**

*should read:*

**Marcello Henrique Nogueira-Barbosa**

Prof. Dr. Edson Marchiori

Editor-in-Chief of **Radiologia Brasileira**

